# Profiles of COVID-19 vaccine hesitancy by race and ethnicity in eastern Pennsylvania

**DOI:** 10.1371/journal.pone.0280245

**Published:** 2023-02-06

**Authors:** Kenya M. Colvin, Kennedy S. Camara, Latasha S. Adams, Adline P. Sarpong, Danielle G. Fuller, Sadie E. Peck, Anthony S. Ramos, Ariana L. Acevedo, Meless A. Badume, Shae-lyn A. Briggs, Tiffany N. Chukwurah, Zanett Davila-Gutierrez, James A. Ewing, Jemimah O. Frempong, Amirah A. Garrett, Steven J. Grampp, Jahasia W. Gillespie, Emmanuel J. Herrera, Shantia M. E. Horsford, Emis J. Maddox, John C. Pelaez, Olivia L. Quartey, Fanny Rodriguez, Luis A. Vasquez, Brian J. Piper, Swathi Gowtham

**Affiliations:** 1 Geisinger Commonwealth School of Medicine, Scranton, Pennsylvania, United States of America; 2 Wilkes University, Wilkes-Barre, Pennsylvania, United States of America; 3 Swarthmore College, Swarthmore, Pennsylvania, United States of America; 4 Temple University, Philadelphia, Pennsylvania, United States of America; 5 Pennsylvania State University, Scranton, Pennsylvania, United States of America; 6 University of Scranton, Scranton, Pennsylvania, United States of America; 7 Susquehanna University, Selinsgrove, Pennsylvania, United States of America; 8 Center for Pharmacy Innovation and Outcomes, Geisinger Precision Health Center, Forty Fort, Pennsylvania, United States of America; 9 Department of Pediatrics, Pediatric Infectious Diseases, Geisinger Medical Center, Danville, Pennsylvania, United States of America; Ahmadu Bello University, NIGERIA

## Abstract

**Background:**

Throughout US history, chronic and infectious diseases have severely impacted minority communities due to a lack of accessibility to quality healthcare and accurate information, as well as underlying racism. These fault lines in the care of minority communities in the US have been further exacerbated by the rise of the COVID-19 pandemic. This study examined the factors associated with COVID-19 vaccine hesitancy by race and ethnicity, particularly among African American and Latinx communities in Eastern Pennsylvania (PA).

**Methods:**

Survey data was collected in July 2021 in Philadelphia, Scranton, Wilkes-Barre, and Hazleton, PA. The 203 participants (38.7% Black, 27.5% Latinx) completed the 28-question survey of COVID-19 vaccination attitudes in either English or Spanish.

**Result:**

Out of the 203 participants, 181 participants met all the inclusion criteria, including completed surveys; of these participants, over three-fifths (63.5%) were acceptant of the COVID-19 vaccine whereas the remainder (36.5%) were hesitant. Binary logistic regression results showed that age, concern for vaccine efficacy, race, knowledge on the vaccine, and belief that the COVID-19 virus is serious significantly influenced COVID-19 vaccine hesitancy. Minorities were more likely to be hesitant toward vaccination (OR: 2.8, 95% CI: 1.1, 6.8) than non-Hispanic whites. Those who believed the COVID-19 vaccine was ineffective (OR: 8.3, 95% CI: 3.8, 18.2), and that the virus is not serious (OR: 8.3, 95% CI: 1.1, 61.8) showed the greatest odds of hesitancy.

**Conclusions:**

Minority status, age less than 45 years, misinformation about seriousness of COVID-19 illness, and concern about vaccine efficacy were contributing factors of COVID-19 vaccine hesitancy. Therefore, understanding and addressing the barriers to COVID-19 vaccination in minority groups is essential to decreasing transmission and controlling this pandemic, and will provide lessons on how to implement public health measures in future pandemics.

## Background

Health disparities are differences in disease prevention measures and health outcomes in socially disadvantaged communities [1]. Socially disadvantaged communities can include certain racial and ethnic groups, LGBTQ+ communities, rural or urban communities, and low socioeconomic areas [1]. Racial and ethnic health disparities have been evident in healthcare in the US for many years. For example, African American and Latinx communities are more likely to suffer from cardiovascular diseases compared to non-Hispanic whites [2]. These disparities have been attributed to several factors including socioeconomic status, levels of education, structural racism, medical mistrust, and lack of quality healthcare access [2–4]. Low socioeconomic status has negatively influenced health outcomes for socially disadvantaged communities. This association is attributed to individuals with a low socioeconomic status lacking health insurance or lacking access to quality healthcare.

This has been further evident in worse health outcomes in socially disadvantaged communities regarding COVID-19. Lower socioeconomic communities have higher prevalence rates of contracting COVID-19 compared to higher socioeconomic groups [5]. The geographical concentration of minorities in urban areas where many work in public service jobs and do not have the option to work from home, may also contribute to the disproportionate, negative impact COVID-19 has had on minority communities across the US [6]. Urban settings are more likely to be densely populated and have limited capacity for social distancing [6]. African American and Hispanic/Latinx communities across the US have experienced higher rates of infection, hospitalization, readmission, and death due to COVID-19 compared to non-Hispanic whites [7–12]. Higher mortality rates amongst these communities may be due to higher comorbidities, such as cardiovascular illnesses, diabetes, and lack of timely access to quality healthcare [2,6]. Unfortunately, the administration of effective outpatient therapies for COVID-19 like monoclonal antibodies has not been equitable by race and ethnicity [13].

When COVID-19 vaccines became available in December 2020, vaccine uptake among these two major ethnic groups remained low in part due to inequities in vaccine distribution across the US [7]. In addition, U.S. African American and Latinx communities displayed high levels of hesitancy for the COVID-19 vaccine in January 2021 [14]. Vaccine hesitancy has been a major, global public health concern for many years, and has gained increasing attention as the world races to end the coronavirus pandemic [7,15]. Vaccine hesitancy exists on a spectrum and is reluctance or complete refusal to receive a vaccine regardless of availability due to a complex interplay of societal and epidemiological factors [16]. Vaccine hesitancy is often context-specific, meaning that individuals who are willing to take one vaccine may be reluctant or refuse to take another [8]. Although vaccine hesitancy is generally well documented, data on vaccine hesitancy in the context of the COVID-19 vaccine is limited and continually evolving as attitudes towards vaccination shift [17,18]. Several national studies conducted in April 2020, May 2020, June 2020, and November 2020 predicted COVID-19 vaccine uptake in the US were published prior to the vaccines becoming available [19,20]. One large, national survey-based study conducted in June 2020 found vaccine hesitancy among African Americans to be 34% and 29% among Hispanics [15].

Since vaccine hesitancy is context-specific, recommended mitigation strategies often include engaging with local communities to collectively develop solutions that address their reluctance toward vaccination [7,8]. Despite several national studies [7,15,20,21], few studies have examined factors driving hesitancy among ethnic minorities by state and even fewer for the state of Pennsylvania (PA) [22]. Our research aimed to identify factors influencing vaccine disparities in eastern PA. Gaining a deeper understanding of these underlying issues may lead to effective, community-specific solutions that promote vaccine uptake. In the first year of the pandemic, African Americans and Latinx communities in PA faced a rate of COVID-19 infection (18% and 21% of cases respectively) greater than their percentage of the state’s population (11% and 7% of the population respectively) [23]. The dynamic nature of the COVID-19 pandemic requires continued monitoring of vaccination uptake rates as this directly impacts the ability to end the pandemic worldwide [7]. The continued hospitalization of unvaccinated patients across the US demonstrates the urgent need to increase vaccination and booster uptake in minority communities [9,24]. The objectives of this study were to outline the major influences on COVID-19 vaccine hesitancy by race, particularly among Latinx and African Americans in Eastern PA and to disseminate accurate vaccine information in the community.

## Materials and methods

### Participants

Data for the participants was collected from a convenience sample in the Philadelphia, Hazleton, Scranton, and Wilkes-Barre areas of Eastern PA. The target population for this study were people ages 18 and older who lived in these areas that had access to information about the vaccine, with a focus on the underrepresented minority population. No sample size calculations were done as the data that was collected was from readily available convenience sample.

### Procedures

#### Study tool

This study was based on collecting survey data from volunteer participants; the data was collected using SurveyMonkey. The survey questions were adopted and modified from several studies [15,25–29]. The surveys were completed on 8^th^ generation iPad, model A2270, 99.9% of the time (n = 201/203). 2 participants preferred to complete the survey on paper.

#### Consent

Investigators obtained verbal consent as well as verbal confirmation of participants being at least 18 years of age prior to survey completion. Each survey team included at least one fluent Spanish-speaking student.

#### Data collection

Members of the Center of Excellence summer program (COE) identified high traffic areas in the cities of interest. Personal protective equipment was worn throughout data collection in accordance with CDC guidelines to ensure the safety of the research team as well as the public. Surveys were filled out by the respondents on the iPads and given back to the research team after completion. Between each respondent, one member of the COE wiped the iPads down with sanitizer wipes. The study was conducted throughout July 2021 using a bilingual script to maintain consistency across sites. Educational materials were developed and distributed with information gathered about the vaccines in the form of fact sheets. In an effort to increase vaccine uptake among the participants, links to resources, including the nearest vaccination location and different community organizations involved in vaccination efforts were also shared with participants who were interested.

The survey and procedures were approved as exempt by the Institutional Review Board of Geisinger (2021–0428).

#### Data analysis

The survey contained 28 questions organized into the following four categories: demographics, attitude towards COVID-19 disease, healthcare, and attitude towards the COVID-19 vaccine (S1 Table). We coded 19 of the 28 questions into dichotomous variables by grouping response choices for each category into two options. Open-ended response questions and questions that were unable to be dichotomized into two distinct groups are not presented here.

Participants who answered less than 10% of the survey were excluded (N = 19). Descriptive analysis was performed separately on each categorical variable to characterize the participants’ socio-demographics (Table 1). For gender, there were three categories: male, female, and non-binary gender. The very small subset of participants who identified as non-binary gender (N = 3) were not included in this analysis given the small number. Therefore, of the 203 initial participants, 19 were excluded due to incomplete survey responses, and 3 participants identifying as non-binary were also excluded. A total of 181 participants were included in the final analysis. Binary logistic regression was performed first to identify which factors had a statistically significant impact on vaccine hesitancy. The results were considered statistically significant if the p-value was less than 0.05. The significant factors were then incorporated into a multinomial logistic regression model. Missing values were removed for the descriptive analyses and both binary and multinomial regression. Multinomial logistic regression was used due to the method’s effectiveness in analyzing multiple categorical variables. Multinomial logistic regression was employed to determine which factors had the greatest influence on vaccine hesitancy. To develop a regression model, 19 of the 28 questions were dichotomized (Table 1). A forward regression was run using vaccine acceptance as the reference category for the dependent variable of vaccine hesitancy. The data analysis was conducted using IBM SPSS Statistics 28. Figures were developed using GraphPad Prism 9.3.1 for Windows.

**Table 1:** Demographics of participants in eastern Pennsylvania completing a survey of COVID-19 vaccination attitudes.

Variable	Percentage of Respondents n (%)	Total (N)
Age		197
Under 45 (18–45)	146 (74.1)	
Over 45 (≥45–65+)	51 (25.9)	
Gender		197
Male	97 (49.2)	
Female	100 (50.8)	
Education Level		197
Secondary	118 (59.9)	
Some High School (No Diploma)	8 (0.04)	
High School Diploma or GED	110 (55.8)	
Post-Secondary	79 (40.1)	
Associate Degree	24 (12.1)	
Bachelor’s Degree	37 (18.8)	
Master’s Degree	14 (0.07)	
Doctorate’s Degree	3 (0.02)	
Professional Degree beyond Bachelor’s	1 (0.01)	
Race		196
Non-Hispanic White	57 (29.4)	
Minority	139 (70.6)	
Black or African American	76 (38.7)	
Hispanic, Latinx, or of Spanish Origin	54 (27.5)	
Asian	9 (4.6)	
American Indian or Alaska Native	1 (0.51)	
Middle Eastern or North African	1 (0.51)	
Native Hawaiian or other Pacific Islander	0 (0)	
Multiethnic	7 (3.5)	
Health Insurance		192
Yes	165 (85.9)	
No	27 (14.0)	
Affected by COVID-19		192
Not Affected	48 (25.0)	
Affected	144 (75.0)	
“If you have not gotten the vaccine, how safe do you think the vaccine is?”		72
Safe	18 (25.0)	
Not safe	54 (75.0)	
“I have concerns that the COVID-19 vaccine is not effective.”		183
Agree	88 (48.1)	
Disagree	95 (51.9)	
“If you do not want to get the vaccine, which answer best describes your reason for not getting the vaccine?”		71
Personal reasons	30 (42.2)	
Distrustful of scientific community	41 (57.7)	
Concerned about getting COVID-19 again		35
Not worried	19 (54.2)	
Worried	16 (45.7)	
Paid sick leave		191
Yes	124 (64.9)	
No	67(35.0)	
History of COVID-19		197
Yes	35 (17.8)	
No	162 (82.1)	
If no history of COVID-19, how worried are you about getting it?		156
Not worried	77 (49.4)	
Worried	79 (50.6)	
“How serious do you think the COVID-19 virus is?”		192
Serious	182 (94.7)	
Not serious	10 (5.21)	
Knowledgeable about the COVID-19 vaccine		186
Knowledgeable	166 (89.2)	
Not Knowledgeable	20 (10.8)	
“Where do you get most of your information about the vaccine?”		183
More than 3 sources	17 (9.3)	
Three or fewer sources	166 (90.7)	
Impact of COVID-19 Information Sources on the community		186
Positive	99 (53.2)	
Negative	87 (46.8)	
“Where do you obtain your information on COVID-19”		184
More than 3 sources	17 (9.2)	
Three or fewer sources	167 (90.8)	
Vaccine Hesitancy		181
Acceptant	115 (63.5)	
Like to get it as soon as possible	6 (3.3)	
I have already gotten it	111 (61.3)	
Hesitant	66 (36.4)	
Never get the vaccine	26 (14.4)	
Only if it is required by the state or employer	8 (4.4)	
Wait and see	32 (17.7)	

COVID-19 vaccine information sources included: My doctor, major news channels, the newspaper, social media, family members, or other. Information sources for the COVID-19 disease included: Social media, local news, CDC, local pharmacies, medical professionals, family/friends, coworkers, or others.

## Results

Table 1 illustrates the demographics of the participants and the 19 of the 28 questions that were dichotomized. Among the participants, 198 (97.5%) individuals filled the survey out in English and 5 (2.5%) in Spanish. The ages of the participants ranged from 18 to over 65. The sample population contained nearly equal amounts of men and women. Over half of the participants identified as African American or Latinx (Table 1). Education level was dichotomized into secondary and post-secondary. Most participants (n = 116, 59.7%) had secondary educational qualifications (some high school to a high school diploma) while post-secondary (associates degree and beyond) accounted for the remaining two-fifths (n = 78, 40.2%). The vast majority (n = 165, 85.9%) of participants had health insurance although one-seventh (n = 27, 14.1%) did not.

**Fig 1:**
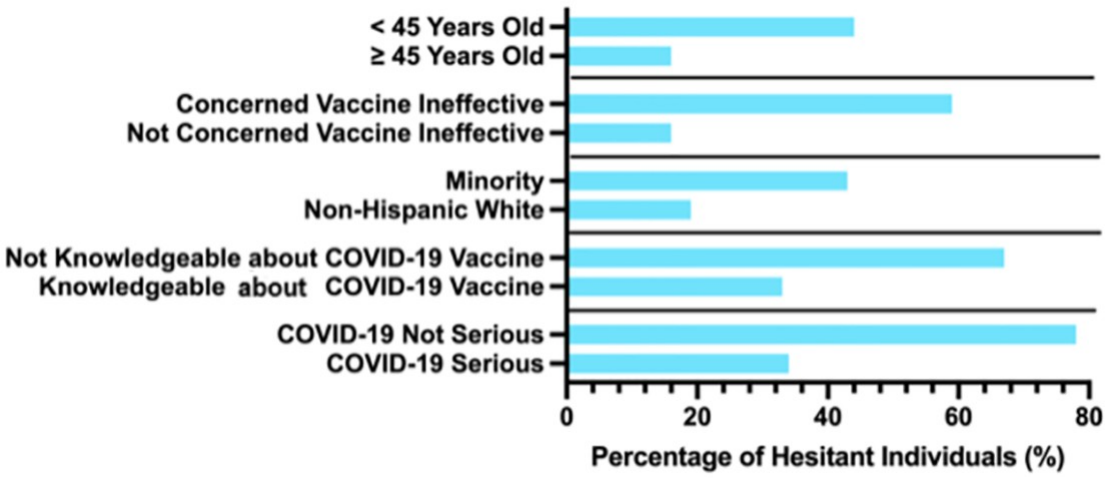
Bar graph of the percentage of hesitant individuals towards COVID-19 vaccine in eastern Pennsylvania with influential factors. Percentage of hesitant individuals towards the COVID-19 vaccine in Eastern Pennsylvania for each factor found to be significant during binary regression (N = 181).

To determine which factors had the most influence on vaccine hesitancy, binary logistic regression was performed for each dichotomized variable in Table 1. When the p-value was less than 0.05, the results were considered statistically significant. The following variables had a statistically significant impact on vaccine hesitancy: younger age (p ≤ 0.001), concern that the vaccine is not effective (p ≤ 0.001), race (p ≤ 0.003), lack of knowledge about the COVID-19 vaccine (p = 0.008), and belief that COVID-19 did not cause serious illness (p ≤ 0.020). S2 Table depicts the most influential factors associated with COVID-19 vaccine hesitancy and the results of the multinomial logistic regression. The multinomial logistic regression took all the significant factors to determine which had the greatest influence on vaccine hesitancy. A total of 181 participants met the inclusion criteria and were used for this data analysis. Overall, 63.5% (n = 115) of participants were found to be acceptant of the COVID-19 vaccine and 36.5% (n = 66) were hesitant. In their responses, individuals who were vaccine hesitant tended to report that “COVID-19 is not serious,” “Concerned the vaccine not effective,” and “Not knowledgeable on the vaccine,” (Fig 1). Although 95% of the respondents deemed COVID-19 as serious, 36.5% of those individuals remained hesitant towards vaccination. Most (90.0%) participants said that they were knowledgeable about the vaccine and the remaining (n = 18, 10.0%) admitted to having little knowledge of the vaccine.

**Fig 2:**
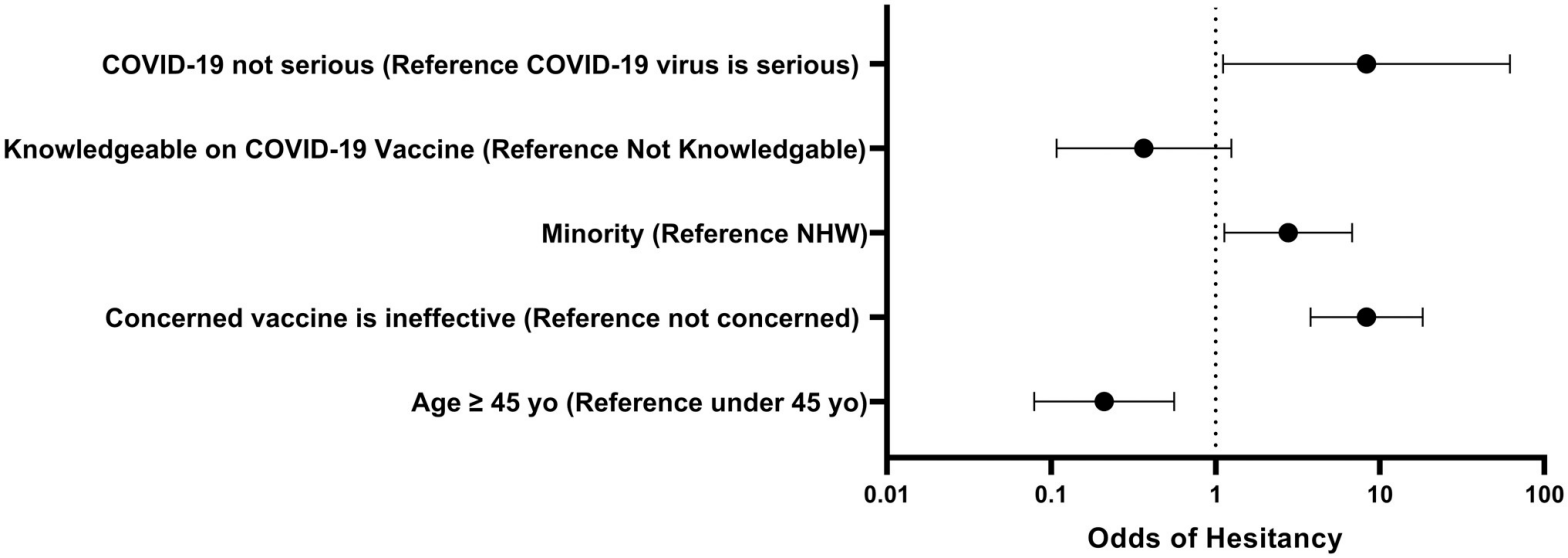
Forest plot of influential factors associated with vaccine hesitancy (S2 Table). N = 181. Individuals who thought COVID-19 was serious, identified as a minority and were under 45 years of age show an increased likelihood of being hesitant toward the COVID-19 vaccine by multinomial regression. NHW—non-Hispanic white Eastern PA sample.

We found that participants ≥ 45 years of age were less likely to be hesitant towards the vaccine (OR: 0.2, 95% CI: 0.1, 0.6) than those under 45. Individuals who agreed that they were concerned that the vaccine is ineffective showed 58.6% hesitancy (OR: 8.3, 95%CI: 3.8, 18.2) and 41.4% were acceptant of the vaccine. Minorities were more likely to show hesitancy—43.4% hesitancy (OR: 2.8, 95% CI: 1.1, 6.8) than non-Hispanic whites (NHW), 19.2% hesitancy. Of those who thought themselves knowledgeable about the vaccine, 66.9% expressed acceptance and 33.1% were hesitant (OR: 0.4, 95% CI: 0.1, 1.3). Knowledgeability about the vaccine was found to be a significant predictor of vaccine hesitancy during the bivariate analysis, but not statistically significant after the multinomial regression was completed. For those who believed the COVID-19 infection is not serious, 22.2% were acceptant of the vaccine, while 77.8% were hesitant (OR: 8.3, 95% CI: 1.1, 61.8). Fig 2 shows the odds of hesitancy for these four statistically significant predictors.

## Discussion

COVID-19 vaccines have shown clear efficacy in reducing the number of hospitalizations and death from severe COVID-19 disease, and therefore vaccine acceptance by all eligible recipients will be the cornerstone of public health intervention [20]. Our focus on assessing the profile of unvaccinated and vaccinated individuals in our communities in eastern Pennsylvania is essential for understanding how to promote COVID-19 vaccine acceptance. Given that disproportionate numbers of Black and Latinx communities were unvaccinated, we sought to assess the barriers to vaccine acceptance by race and ethnicity in our communities. We analyzed a convenience sample of 181 respondents amongst various areas of Eastern PA. Our findings from the summer of 2021 revealed only moderate COVID-19 vaccine acceptance (63.5%). The remaining 36.5% represent the respondents who identified as vaccine hesitant. In 2021 published literature, vaccine hesitancy accounted for 26.3% of adult Americans across a final pool of 107,841 participants from 13 studies [13]. Although over three-fifths of the study participants showed vaccine acceptance, there was still a significant amount of vaccine hesitancy that needed to be addressed. Our results indicate that the following factors were significant among the vaccine-hesitant: concern that vaccine was not effective, not being knowledgeable about COVID-19 vaccine, and that the COVID-19 illness is not serious. Other significant factors included age and race. Our findings of contributors to vaccine hesitancy were similar to other studies [15,19], including concern about COVID-19 vaccine efficacy. Other studies have reported the profiles of hesitant individuals included female gender, larger household size, those with children at home, and political party affiliation [15,19,21].

Contrary to many other studies [7,15,16,19], the results of our binary regression comparing education level, secondary versus post-secondary education, suggest that education level had no impact on vaccine hesitancy. The results potentially suggest that other factors contributed to our sample’s vaccine acceptance or hesitance. Similar to other published literature [7,15,16,19], our findings identified vaccine hesitancy being greater amongst minority populations compared to non-Hispanic whites. These results suggest that public health messaging regarding COVID-19 vaccine acceptance should prioritize and focus on promoting COVID-19 vaccine equity in minority groups, regardless of education status. Members of these communities should have ready access to accurate information about vaccine safety and efficacy, as well as the devasting effects that COVID-19 illness has disproportionally wrought on these communities.

Consistent with the results from a previous study, vaccine acceptance revealed an association with knowledge about the COVID-19 vaccine [16]. In our data set, among those who reported not being knowledgeable about the COVID-19 vaccine, a majority, 66.7% were also vaccine-hesitant. In contrast, of those who are acceptant of the COVID-19 vaccine, a majority, 66.9% reported being knowledgeable about the vaccine. These findings of hesitancy, therefore, can result from a lack of understanding concerning the COVID-19 vaccine, or be due to the poor sources of the information that the subjects relied upon. Knowledge about the COVID-19 vaccine intersected with trust reporting high confidence levels in healthcare professionals [20]. Whether or not an individual trusts the source of information can influence how receptive one is to the information. Rapid and ever-changing developments regarding COVID-19 and its vaccine were found to have contributed to individuals’ mistrust of outsourced information [8]. The legacy of Tuskegee, and Marion J Simms hangs over, and contributes to the mistrust of medical community in the African-American community in particular. Lack of that trust and reliable sources from an individual’s perspective can explain vaccine hesitancy, particularly in minority populations. More research is needed to regain this trust and to find improved ways to disseminate correct and relevant information about the COVID-19 vaccines and boosters.

Local news and public health resources (e.g., the Centers for Disease Control and Prevention) are various ways the general population receives information concerning COVID-19 and its vaccine [7,16]. The following recommendations are offered to make information concerning COVID-19 vaccines better received and promote vaccine acceptance. Community-based talks and direct personal contact, particularly using trusted community leaders and representatives, can encourage those who are hesitant about vaccine uptake. Contrary to the inaccurate information being disseminated across various media and social media platforms [7], accurate information that is respectfully delivered by trusted messengers must take its place. Studies have concluded that media and scientific outlets have begun to release accurate information to enlighten the general public about the COVID-19 vaccine to mitigate vaccine hesitancy [9,15]. Informational sources concerning COVID-19 and its vaccine should be explicit and clear. Information needs to be supported with medical evidence that can be easily understood and championed by trusted experts and trusted members or leaders within the communities of interest. Implementing inclusive programs for informational purposes, including live seminars for community groups, can alleviate those hesitant and promote acceptance [8].

Although this investigation involved responding quickly to a dynamic public health situation, the observed results are limited by selection bias due to convenience sampling, only a moderate sample size, and occasional missing responses on select variables. Data collection through self-reporting reflects another limitation as self-assessment of COVID-19 knowledge can introduce biases. Other limitations include the few native Spanish-speaking participants in our sample and access to unvaccinated individuals. Most of the population surveyed had already received the vaccine or were receptive to getting it. We cannot discount that some participants holding strong anti-vaccine beliefs were less likely to participate in this voluntary study, again leading to selection bias, and therefore may not be entirely generalizable for all vaccine hesitant individuals [21]. However, through our study, we were able to reach many individuals within our local Black and Latinx communities and provide them with real-time valuable vaccination information, including nearest the vaccination centers for themselves or their family members. Community-based efforts such as those demonstrated in our study will be needed to improve vaccine uptake. Future studies looking at inequities in vaccine uptake should seek to include sizable proportions of unvaccinated individuals and recruit Spanish-speaking participants by using bilingual research assistants and interpreters [21]. Future investigations that include an appreciable number of unvaccinated, or those that have not received a booster dose, may further clarify the overall profile of under-vaccinated minorities, allowing for the development of a more nuanced strategy to address their concerns, and encourage vaccine and booster acceptance among all eligible recipients [30]. While interest regarding COVID-19 illness and its vaccine is waning in the general public and medical community at large, the lessons learnt from studies such as ours will still be relevant as we prepare to battle the next pandemic.

## Conclusion

In this local community-based study on COVID-19 vaccine hesitancy in eastern Pennsylvania, the following variables impacted vaccination hesitancy: younger age, concern about vaccine effectiveness, race, lack of knowledge about the COVID-19 vaccine, and perceived lack of severity of COVID-19 illness. While additional research may be needed to further understand the complexities behind COVID-19 vaccine hesitancy, this investigation enables better-targeted outreach to local communities to unvaccinated or under-vaccinated individuals. In a world where the information is ever-changing and where misinformation is prevalent and readily accessible, public health and infectious diseases research regarding addressing root causes of mistrust of medical sources is imperative to eliminate the vaccination disparities in our current collective response to the COVID-19 pandemic and to effectively face any challenges from pandemics in the future.

## Supporting information

S1 Table Survey questions in English with corresponding Spanish questions. A total of 28 questions were asked and consisted of four categories: Demographics (5), COVID-19 (9), Healthcare (2), and COVID-19 vaccine (11).
(DOCX)

S2 Table Most influential predictors of COVID-19 vaccine hesitancy from multinomial logistic regression model. (DOCX)

## References

[1] Health Disparities | DASH | CDC. Published November 24, 2020. Accessed September 6, 2021. https://www.cdc.gov/healthyyouth/disparities/index.htm.

[2] Clarke AR, Goddu AP, Nocon RS, Stock NW, Chyr LC, Akuoko JAS, et al. Thirty years of disparities intervention research: What are we doing to close racial and ethnic gaps in health care? *Med Care*. 2013;51(11): doi: 10.1097/MLR.0b013e3182a97ba3 24128746 PMC3826431

[3] Njoku A, Joseph M, Felix R. Changing the narrative: Structural barriers and racial and ethnic inequities in COVID-19 vaccination. *Int J Environ Res Public Health*. 2021;18(18):9904. doi: 10.3390/ijerph18189904 34574827 PMC8470519

[4] Towne SD Jr.. Socioeconomic, geospatial, and geopolitical disparities in access to health care in the US 2011–2015. *Int J Environ Res Public Health*. 2017; 14(6):573. doi: 10.3390/ijerph14060573 28555045 PMC5486259

[5] Thakur N, Lovinsky-Desir S, Bime C, Wisnivesky JP. The structural and social determinants of the racial/ethnic disparities in the U.S. COVID-19 pandemic. What’s our role? *Am J Respir Crit Care Med*. 2020;202(7):943–949. doi: 10.1164/rccm.202005-1523PP 32677842 PMC7528789

[6] Webb Hooper M, Nápoles AM, Pérez-Stable EJ. COVID-19 and racial/ethnic disparities. *JAMA*. 2020;323(24):2466–2467. doi: 10.1001/jama.2020.8598 32391864 PMC9310097

[7] Hildreth JEK, Alcendor DJ. Targeting COVID-19 vaccine hesitancy in minority populations in the US: Implications for herd immunity. *Vaccines (Basel)*. 2021;9(5):489. doi: 10.3390/vaccines9050489 34064726 PMC8151325

[8] Strully KW, Harrison TM, Pardo TA, Carleo-Evangelist J. Strategies to address COVID-19 vaccine hesitancy and mitigate health disparities in minority populations. *Front Public Health*. 2021;9:645268. doi: 10.3389/fpubh.2021.645268 33968884 PMC8102721

[9] Bohrn MA, Benenson R, Bush CM, Bell T, Black C, Doan B, et al. Demographics and clinical characteristics of adult patients hospitalized due to COVID-19 in a rural/suburban integrated health system in southcentral Pennsylvania, March through May 2020. *Open Forum Infect Dis*. 2021;8(10). doi: 10.1093/ofid/ofab132 34631913 PMC7989167

[10] Boland MR, Liu J, Balocchi C, Meeker J, Bai R, Mellis I, et al. Association of neighborhood-level factors and COVID-19 infection patterns in Philadelphia using spatial regression. *AMIA Jt Summits Transl Sci Proc*. 2021:545–554. 34457170 PMC8378638

[11] Freeman MC, Gaietto K, DiCicco LA, Rauenswinter S, Squire JR, Aldewereld Z, et al. A comprehensive clinical description of pediatric SARS-CoV-2 infection in western Pennsylvania. Preprint. *medRxiv*. 2020;2020.12.14.20248192. doi: 10.1101/2020.12.14.20248192 33354687 PMC7755149

[12] Nimgaonkar V, Thompson JC, Pantalone L, Cook T, Kontos D, McCarthy AM, et al. Racial disparities in 30-day outcomes following index admission for COVID-19. *Front Med (Lausanne)*. 2021;8:750650. doi: 10.3389/fmed.2021.750650 34796186 PMC8592899

[13] Wiltz JL, Feehan AK, Molinari NM, Ladva CN, Truman BI, Hall J, et al. Racial and ethnic disparities in receipt of medications for treatment of COVID-19—United States, March 2020–August 2021. *MMWR Morb Mortal Wkly Rep* 2022;71(3):96–102. doi: 10.15585/mmwr.mm7103e1 35051133 PMC8774154

[14] Kricorian K, Turner K. COVID-19 vaccine acceptance and beliefs among Black and Hispanic Americans. *PLoS One*. 2021;16(8):e0256122. doi: 10.1371/journal.pone.0256122 34428216 PMC8384224

[15] Khubchandani J, Sharma S, Price JH, Wiblishauser MJ, Sharma M, Webb FJ. COVID-19 vaccination hesitancy in the United States: A rapid national assessment. *J Community Health*. 2021;46(2):270–277. doi: 10.1007/s10900-020-00958-x 33389421 PMC7778842

[16] Kumar D, Chandra R, Mathur M, Samdariya S, Kapoor N. Vaccine hesitancy: Understanding better to address better. *Isr J Health Policy Res*. 2016;5:2. doi: 10.1186/s13584-016-0062-y 26839681 PMC4736490

[17] Dubé E, MacDonald NE. How can a global pandemic affect vaccine hesitancy? *Expert Rev Vaccines*. 2020;19(10):899–901. doi: 10.1080/14760584.2020.1825944 32945213

[18] Webb Hooper M, Nápoles AM, Pérez-Stable EJ. No populations left behind: Vaccine hesitancy and equitable diffusion of effective COVID-19 vaccines. *J Gen Intern Med*. 2021;36(7):2130–2133. doi: 10.1007/s11606-021-06698-5 33754319 PMC7985226

[19] Khubchandani J, Macias Y. COVID-19 vaccination hesitancy in Hispanics and African-Americans: A review and recommendations for practice. *Brain Behav Immun Health*. 2021;15:100277. doi: 10.1016/j.bbih.2021.100277 34036287 PMC8137342

[20] Malik AA, McFadden SM, Elharake J, Omer SB. Determinants of COVID-19 vaccine acceptance in the US. *EClinicalMedicine*. 2020;26:100495. doi: 10.1016/j.eclinm.2020.100495 32838242 PMC7423333

[21] Piper BJ, Sanchez BV, Madera JD, Sulzinski MA. Profiles of US Hispanics unvaccinated for COVID-19. *J Racial Ethn Health Disparities*. 2022;1–7. doi: 10.1007/s40615-022-01245-2 35107819 PMC8809210

[22] Fernández-Penny FE, Jolkovsky EL, Shofer FS, Hemmert KC, Valiuddin H, Uspal JE, et al. COVID-19 vaccine hesitancy among patients in two urban emergency departments. *Acad Emerg Med*. 2021;28(10):1100–1107. doi: 10.1111/acem.14376 34403539 PMC8441923

[23] Treglia D, Addo M, Cusack M, Culhane D. Understanding racial and ethnic disparities in health outcomes and utility insecurity resulting from Covid-19. Community Legal Services of Philadelphia. March 25, 2021. Accessed February 3, 2022. https://clsphila.org/wp-content/uploads/2021/03/CLS_UtilityReport_20200324.pdf.

[24] COVID-19 State Profile Report—Pennsylvania | HealthData.gov. Accessed August 18, 2021. https://healthdata.gov/Community/COVID-19-State-Profile-Report-Pennsylvania/tkdp-r3p8/.

[25] Lucia VC, Kelekar A, Afonso NM. COVID-19 vaccine hesitancy among medical students. *J Public Health (Oxf)*. 2020. doi: 10.1093/pubmed/fdaa230 33367857 PMC7799040

[26] Kreps S, Prasad S, Brownstein JS, Hswen Y, Garibaldi BT, Zhang B, et al. Factors associated with US adults’ likelihood of accepting COVID-19 vaccination. *JAMA Netw Open*. 2020;3(10): e2025594. doi: 10.1001/jamanetworkopen.2020.25594 33079199 PMC7576409

[27] Quinn SC, Jamison A, Freimuth FS, An J, Hancock GR, Musa D. Exploring racial influences on flu vaccine attitudes and behavior: Results of a national survey of African American and White adults. *Vaccine*. 2017;35(8):1167–1174. doi: 10.1016/j.vaccine.2016.12.046 28126202 PMC5839483

[28] Center of Disease Control and Prevention. National Center for Chronic Disease Prevention and Health Promotion. Demographic questions [Internet] US. [cited 2021 May]. https://www.cdc.gov/workplacehealthpromotion/tools-resources/pdfs/nhwp_demographics_survey.pdf.

[29] Center for Disease Control and Prevention. National Health Interview Survey. National Center for Health Statistics. [Internet]. [cited 2021 May]. https://www.cdc.gov/nchs/nhis/2020nhis.htm.

[30] FDA Office of Media Affairs. FDA authorizes Pfizer-BioNTech COVID-19 vaccine for emergency use in children 5 through 11 years of age. October 29, 2021. [cited2/4/2021]. https://www.fda.gov/news-events/press-announcements/fda-authorizes-pfizer-biontech-covid-19-vaccine-emergency-use-children-5-through-11-years-age.

