# Supplementary material for: Profiles of COVID-19 vaccine hesitancy by race and ethnicity in eastern Pennsylvania
Source: PLoS One. 2023 Feb 6;18(2):e0280245. doi: 10.1371/journal.pone.0280245 (PMC9901750; doi:10.1371/journal.pone.0280245)
Supplement: S1 Table — A total of 28 questions were asked and consisted of four categories: Demographics (5), COVID-19 (9), Healthcare (2), and COVID-19 vaccine (11). (DOCX) [file pone.0280245.s001.docx]

**Supplemental Table 1.** Survey questions in English with corresponding Spanish questions. A total of 28 questions were asked and consisted of four categories: Demographics (5), COVID-19 (9), Healthcare (2), and COVID-19 vaccine (11).

| Question Type | Question number | English Survey Questions | Spanish Survey Questions |
| --- | --- | --- | --- |
| Demographics | 1 | County of residence. | Lugar de residencia. |
|  | 2 | Gender: Pick one. | Género: elegir uno. |
|  | 3 | Explain your answer for question 2. | Elabore más. |
|  | 4 | Race/Ethnicity: Select all that apply to you. | Etina/Raza: |
|  | 5 | Age in Years: Pick one. | Edad en años: elegir uno. |
|  | 6 | What is your highest level of education? | ¿Cuàl es tu grado más alto de educación? |
| COVID-19 | 7 | Have you ever had COVID-19? | ¿Alguna vez has tenido COVID-19? |
|  | 8 | If yes, are you worried about getting it again? | Si pusistes que si en la respuesta anterior, estás preocupado(a) de obtener el virus otra ves? |
|  | 9 | If no, how worried are you about getting it? | Si pusiste que no en la respuesta anterior, estás preocupado(a)? |
|  | 10 | Do you have paid sick leave in case you get sick? | Por si acaso te enfermas, tienes tiempo libre pagado por causa directa a la enfermedad? |
| Healthcare | 11 | Do you have health insurance or Medicaid/Medicare in case you get sick? | Tienes seguro de salud, como Medicare y Mediciaid, en caso de una enfermedad? |
| COVID-19 | 12 | To what extent would you say that you have been affected by COVID-19? | A qual extremo COVID-19 te ha afectado? |
|  | 13 | How serious do you think the COVID-19 virus is? | ¿Qué tan serio crees que es el COVID-19? |
|  | 14 | Where do you obtain your information on COVIDE-19? | ¿Por dónde obtienes tu información sobre el COVID-19? |
|  | 15 | Explain your answer to question 14. | Elabore más. |
|  | 16 | What impact do you believe these sources (listed above) have had on your community? | ¿Qué tipo de impacto crees que estas procedencia de información tienen en tu comunidad? |
|  | 17 | Personally, what stage do you think we are in the timeline of the COVID-19 pandemic? | Personalmente, en cuàl etapa crees que estamos en el COVID-19? |
| COVID-19 vaccine | 18 | How knowledgeable would you say you are about the COVID-19 vaccine? | Cuanto conocimiento dirías que tienes sobre la vacuna contra COVID-19? |
|  | 19 | Where do you get most of your information about the vaccine? | De donde obtiene la mayoría de información sobre la vacuna? |
|  | 20 | Explain your answer to question 19. | Elabore más. |
|  | 21 | Agree or Disagree: I have concerns that the COVID-19 vaccine is not effective. | Yo tengo duda sobre la efectividad de la vacuna. |
|  | 22 | COVID-19 vaccine is now available to protect people from getting the infection. If you had the opportunity to get the vaccine, would you... | La vacuna contra el COVID es accesible ahora para prevenir el contacto con la infección. Si usted tuviera la oportunidad de recibir la vacuna, usted... |
|  |  |  |  |
|  | 23 | If you have not yet gotten the vaccine, how safe do you think the vaccine is? | Si no has recibido la vacuna, ¿qué tan segura crees que es? |
|  | 24 | Which of the following best explains why you chose to be vaccinated? | ¿Cuál de las razones más explica porque te vacunastes? |
|  | 25 | Explain your answer to question 24. | Elabore más. |
|  | 26 | If you do not want to get the vaccine, which answer best describes your reason for not getting the vaccine? | Si no quieres la vacuna, ¿ cuál contestación resuena con tu explicación? |
|  | 27 | Explain your answer to question 26. | Elabore más. |
|  | 28 | If you want the vaccine, do you know where to get the vaccine? | Si quieres la vacuna, ¿sabes adonde te la puedes poner? |
