# Supplementary material for: Profiles of COVID-19 vaccine hesitancy by race and ethnicity in eastern Pennsylvania
Source: PLoS One. 2023 Feb 6;18(2):e0280245. doi: 10.1371/journal.pone.0280245 (PMC9901750; doi:10.1371/journal.pone.0280245)
Supplement: S2 Table — (DOCX) [file pone.0280245.s002.docx]

**Supplemental Table 2**. Most influential predictors of COVID-19 Vaccine Hesitancy from multinomial logistic regression model.

| **Factors** |  | **Hesitant**  **n (%)** | **Acceptant**  **n (%)** | **OR** | **95% CI** | ***p*-value** |
| --- | --- | --- | --- | --- | --- | --- |
| Age | Over 45  Under45 | 8(16.3)  58(43.9) | 41(83.7)  74(56.1) | .210 | [.079,.559] | .002 |
| Vaccine Ineffectiveness | Agree  Disagree | 51(58.6)  15(16.0) | 36(41.4)  79(84.0) | 8.29 | [3.78,18.2] | <.001 |
| Race | Minority  Non-Hispanic White | 56(43.4)  10(19.2) | 73(56.6)  42(80.8) | 2.77 | [1.13,6.79] | .026 |
| Knowledgeable about COVID-19 Vaccine | Knowledgeable  Non-knowledgeable | 54(33.1)  12(66.7) | 109(66.9)  6(33.3) | .366 | [.108,1.25] | .108 |
| COVID-19 Disease  Seriousness | Not serious  Serious | 7(77.8)  59(34.3) | 2(22.2)  113(65.7) | 8.28 | [1.11, 61.8] | .039 |
